# Presentation, management, and outcomes of STEMI in Egypt: results from the European Society of Cardiology Registry on ST elevation myocardial infarction

**DOI:** 10.1186/s43044-020-00069-x

**Published:** 2020-07-01

**Authors:** Sameh Shaheen, Ahmad Wafa, Mostafa Mokarab, Basem Zareef, Ahmed Bendary, Tarek Abdelhameed, Ahmad Rashwan, Mohamad Seleem, Magdy Elmasry, Yaser Abdelhady, Gomaa Abdelrazik, Amr Ibrahim, Mohamad Ghareeb, Khalid Aly, Mahmoud Saraya, Moheb Wadie, Mahmoud Youssef

**Affiliations:** 1 Cardiology Department, Ain Shams University Hospitals, Ain Shams University, Cairo, Egypt; 2 Cardiology Department; Mansoura University Hospital, Mansoura Faculty of Medicine, Mansoura, Egypt; 3 Cardiology Department, Bab Elsheria and Alhosien University Hospitals, Al-Azhar Faculty of Medicine, Cairo, Egypt; 4 Cardiology Depatrtment, National Heart Institute, Giza, Egypt; 5 Cardiology Department, Banha University Hospital, Banha Faculty of Medicine, Banha, Egypt; 6 Cardiology Department, Assiut University Hospitals, Assiut University, Assiut, Egypt; 7 Cardiology Department, Fayoum General Hospital, Fayoum, Egypt; 8 Cardiology Department, National Heart Institute, Giza, Egypt; 9 Cardiology Department, Tanta University Hospital, Tanta University, Tanta, Egypt; 10 Cardiology Department, Bani Sweif University Hospital, Bani Sweif University, Bani Sweif, Egypt; 11 Cardiology Department, Fayoum University Hospital, Fayoum University, Fayoum, Egypt; 12 Cardiology Department, Helwan University Hospital, Helwan University, Badr City, Egypt; 13 Cardiology Department, Nasr City Insurance Hospital, Cairo, Egypt; 14 Cardiology Department, Cairo University Hospitals, Kasr Alainy Faculty of Medicine, Cairo, Egypt

**Keywords:** STEMI, Registry, Egypt, Primary PCI, Thrombolytic, Reperfusion, Networks

## Abstract

**Background:**

Apart from few small single-center studies, there are limited data about STEMI patients in Egypt. Nineteen Egyptian centers (with and without PCI facilities) participated in this registry with 1356 patients who were compared to 7420 patients from other ESC countries. The aims of this study were to describe the characteristics of patients with STEMI, to assess STEMI management patterns particularly the current use of reperfusion therapies, to evaluate the organization of STEMI networks across Egypt, to evaluate in-hospital patient outcome, and to compare Egyptian patients with other ESC countries.

**Results:**

Compared to other ESC countries, Egyptian patients were younger (mean age 55.4 ± 11.3 vs. 62.9 ± 12.4; *p* < 0.001 and 4.36% vs. 19.41%% were ≥ 75 years old; *p* < 0.001) with fewer females (18.44% vs. 25.63%; *p* < 0.001). Egypt had longer median time between symptoms onset and first medical contact: 120.0 (60.0; 240.0) vs.100.0 (50.0; 240.0) *p* < 0.001. Self-presentation rather than EMS presentation was the mode of admission in 86.06% in Egypt vs. 25.83% in EU countries (*p* < 0.001). On qualifying ECG, anterior STEMI was in 57.08% in Egypt vs. 45.98% in other countries (*p* < 0.001). Initial reperfusion therapy was 49.12%, 43.07%, and 7.26% for primary PCI, thrombolytic therapy and no reperfusion in Egypt vs. 85.42%, 7.26%, and 7.82% for EU countries, respectively. In-hospital mortality was 4.65% in Egypt vs. 3.50% in other countries *p* 0.040 and was 18.87% in no reperfusion vs. 2.10% in primary PCI vs. 4.97% in thrombolysis (*p* < 0.001) among Egyptians. Patients were discharged on aspirin in 98.61%, clopidogrel in 91.07%, ticagrelor in 7.31%, DAPT in 97.69%, beta blockers in 82.83%, ACE inhibitors in 84.76%, MRAs in 10.01%, and statins in 99.77%.

**Conclusion:**

Compared to other ESC countries, Egyptian STEMI patients were younger, more frequently current smokers and diabetics, and had longer time between symptoms onset and first medical contact with more self-presentation rather than EMS presentation. Thrombolytic therapy is still a common reperfusion therapy in Egypt while primary PCI was offered to half of the patients. In-hospital mortality was significantly higher in Egypt and was highest among no reperfusion patients and lowest among PPCI patients.

## Background

Mortality due to CVD in Egypt is one of the highest compared to other countries in the region and worldwide [1].

Apart from a small number of single center studies there are limited descriptive data about STEMI patients in Egypt [2–4]. The lack of data on the clinical characteristics of patients, hospital practice, and treatment patterns as well as the impact of management on outcomes for STEMI in Egypt underscore the need for a national registry database.

## Methods

The European Society of Cardiology ACCA-EAPCI Registry on ST elevation myocardial infarction is a Joint initiative of the Acute Cardiovascular Care Association (ACCA) and the European Association of Percutaneous Cardiovascular Interventions (EAPCI) of the European Society of Cardiology. It is a registry to evaluate the treatment of STEMI across Europe and the Mediterranean countries. It is a general, prospective, multicenter, and observational registry. The ESC-STEMI registry study design has been described in detail in a recent publication [5].

The EURObservational Research Programme (EORP) department at the European Heart House coordinated the project operationally, provided support to the participating centers, and guided the methodological aspects of the survey. The database was stored and analyzed at the European Heart House.

The Egyptian society of cardiology, being a member of the ESC, participated in this registry with data from 19 Egyptian centers. Site selection aimed at centers of different levels of complexity and in different geographic regions in order to obtain a sample representative of Egyptian population. Onsite PCI capability was present in 15/17 centers (88.24%), onsite cardiac surgery in 11/17 centers (64.71%), and 8/16 (50.00%) reported that primary PCI was their usual treatment for STEMI patients. The median number (Q1; Q3) of beds in these centers was 91.5 (35.0; 325.0), MI volume/year was 500.0 (250.0;720.0), total PCI/year was 800.0 (400.0; 2000.0), and total primary PCI was 220.0 (90.0; 350.0). Local audits were performed in randomly selected centers to check compliance with the protocol and review consecutiveness and quality of data.

The target population was patients with chest pain or equivalent symptoms of more than 20 min duration within the last 24 h prior to admission to hospital and ST segment elevations or LBBB in the diagnostic ECG. Each center was asked to enroll up to 60 consecutive patients. Egypt participated with 1356 (15.45%) patients who will be compared with 7420 patients from other EU or Mediterranean countries. Patient recruitment started from March 2016 to February 2018.

The aims of this study were to describe the demographic, clinical, and biological characteristics of patients with STEMI admitted to a representative setting of cardiology centers in EGYPT, to assess the organization of STEMI management across Egypt, to evaluate how STEMI ESC guidelines were adopted, to evaluate in-hospital patient outcome, and to compare Egyptian patients with those from other participating countries.

Continuous variables were reported in means (standard deviation) or as median and interquartile range (IQR) when skewed. Categorical variables were reported as percentages and compared using the *χ2* test. Continuous variables were compared by the Mann–Whitney *U* test. Kruskal–Wallis test was used when more than two groups were compared. A *p* value of < 0.05 was considered statistically significant. All tests were two-sided.

## Results

### Patients’ characteristics: Table 1

Compared to other countries, Egyptian patients were younger (mean age 55.4 ± 11.3 vs. 62.9 ± 12.4; *p* value < 0.001 and patients ≥ 75 years old were 4.36% vs. 19.41%; *p* value < 0.001) with fewer females (18.44% vs. 25.63%; *p* value < 0.001). Furthermore, fewer Egyptian patients had history of myocardial infarction (7.95% vs. 12.66%; *p* value < 0.001), chronic heart failure (2.07% vs. 11.51%; *p* value < 0.001), Stroke/TIA (3.99% vs. 5.55% *p* value 0.018), PCI (7.10% vs. 10.43% *p* value < 0.001), CABG (0.81% vs. 1.52% *p* value 0.043), atrial fibrillation (1.55% vs. 5.32% *p* value < 0.001), peripheral vascular disease (0.89% vs. 5.89% *p* value < 0.001), and malignancy (0.67% vs. 2.60%, *p* value < 0.001) but no significant difference for patients on dialysis (0.44% vs. 0.26% *p* value 0.265). On the other hand, Egyptian patients had higher prevalence of traditional risk factors such as current smoking (59.05% vs. 42.81%; *p* value < 0.001), diabetes mellitus (40.79% vs. 21.95%, *p* value < 0.001), BMI ≥ 30 kg/m (34.34% vs. 25.90%; *p* value < 0.001), LDL cholesterol (mg/dL) (134.8 ± 43.1 vs. 112.4 ± 40.3; *p* value < 0.001), plasma glucose (mg/dL) (180.8 ± 80.2 vs. 143.6 ± 67.0; *p* value < 0.001) but less history of hypertension (37.23% vs. 52.65%, *p* value < 0.001)

**Table 1:** Patients baseline clinical characteristics; Egypt vs. other ESC countries

	Egypt (*n* = 1356)	Other ESC countries (*n* = 7420)	*p* value
Age (years) median (Q1; Q3)	55.0 (48.0;63.0)	62.0 (54.0;71.0)	< 0.001 (S)
Age ≥ 75 years	59/1354 (4.36%)	1433/7381 (19.41%)	< 0.001(S)
Female	250/1356 (18.44%)	1902/7420 (25.63%)	< 0.001(S)
BMI ≥ 30 kg/m^2^	444/1293 (34.34%)	1809/6984 (25.90%)	< 0.001(S)
Previous myocardial infarction	107/1346 (7.95%)	865/6830 (12.66%)	< 0.001(S)
Previous angina	303/1345 (22.53%)	1645/6746 (24.38%)	0.146(NS)
Chronic heart failure	28/1350 (2.07%)	838/7283 (11.51%)	< 0.001(S)
Previous stroke/TIA	54/1354 (3.99%)	409/7367 (5.55%)	0.018(S)
Previous PCI	96/1353 (7.10%)	769/7370 (10.43%)	< 0.001(S)
Previous CABG	11/1355 (0.81%)	112/7389 (1.52%)	0.043(S)
Atrial fibrillation	21/1355 (1.55%)	392/7366 (5.32%)	< 0.001(S)
Peripheral vascular disease	12/1342 (0.89%)	411/6979 (5.89%)	< 0.001(S)
Current smoker	799/1353 (59.05%)	3097/7234 (42.81%)	< 0.001(S)
Diabetes mellitus	547/1341 (40.79%)	1615/7357 (21.95%)	< 0.001(S)
Hypercholesterolemia	141/1111 (12.69%)	2848/6333 (44.97%)	< 0.001(S)
Treated hypertension	503/1351 (37.23%)	3833/7280 (52.65%)	< 0.001(S)

### Hospital admission process (Fig. 1)

Median time (Q1; Q3) in minutes between symptoms onset and call for medical help in Egypt was 85.0 (30.0; 210.0) vs. 74.5 (30.0; 210.0) in other countries (*p* value 0.039) while the median time between symptoms onset and first medical contact was 120.0 (60.0; 240.0) vs.100.0 (50.0; 240.0) (*p* value < 0.001). In Egypt, compared to other countries, ER staff was the first medical contact (81.42% vs. 24.62%; *p* value < 0.001), self-presentation was the mode of admission (86.06% vs. 25.83%; *p* value < 0.001) and a non PCI capable center was the first hospital receiving STEMI patients (42.04% vs. 26.99%; *p* value < 0.001)

**Fig. 1:**
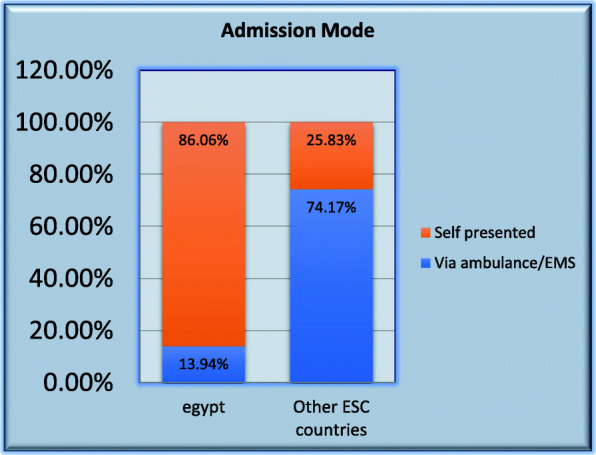
Admission mode in Egypt vs. other countries

### Patient presentation and initial assessment (Table 2)

Patients admitted after an out of hospital cardiac arrest in Egypt was 2.51% vs. 5.49% in EU countries *p* value < 0.001. On qualifying ECG, anterior STEMI was in 57.08% in Egypt vs. 45.98% in other countries (*p* value < 0.001), atrial fibrillation was in 3.47% vs. 6.20% (*p* value < 0.001), and heart rate (mean ± SD) was 84.8 ± 19.4 vs. 78.4 ± 19.6 (*p* value < 0.001). Killip class IV was in 2.88% in Egypt vs. 4.27% in other countries (*p* value < 0.001), mechanical ventilation was used in 4.06% in Egypt vs. 5.67% in other countries (*p* value 0.016), therapeutic hypothermia was used in 1.40% in Egypt vs. 2.14% in other countries (*p* value 0.078), and therapeutic hypothermia when used was external cooling pads/blankets/wraps in 100.00% in Egypt vs. 11.41% in other countries (*p* value < 0.001).

**Table 2:** Presentation and initial assessment; Egypt vs. other countries

	Egypt (*n* = 1356)	other countries (*n* = 7420)	*p* value
Anterior STEMI on qualifying ECG	774/1356 (57.08%)	3205/6971 (45.98%)	< 0.001(S)†
AF on qualifying ECG	47/1356 (3.47%)	460/7420 (6.20%)	< 0.001(S)
HR on qualifying ECG median (Q1; Q3)	85.0 (70.0;100.0)	77.0 (65.0; 90.0)	< 0.001 (S)
SBP at first presentation median (Q1; Q3)	120.0 (110.0;140.0)	135.0 (120.0; 150.0)	< 0.001 (S)
Killip class IV at first presentation	39/1356 (2.88%)	316/7397 (4.27%)	< 0.001(S)
LDL cholesterol (mg/dL) mean (±Std)	134.8 (± 43.1)	112.4 (± 40.3)	< 0.001 (S)
Glucose plasma level (mg/dL) mean (±Std)	180.8 (± 80.2)	143.6 (± 67.0)	< 0.001 (S)

### STEMI reperfusion pattern (Fig. 2)

Intended treatment for STEMI in Egypt vs. other countries was primary PCI in 50.59% vs. 85.50%, thrombolysis in 43.14% vs. 5.55%, and no reperfusion in 6.05% vs. 4.72%. Actual initial reperfusion in Egypt vs. other countries: primary PCI in 49.12% vs.85.42%, thrombolytic therapy 43.07% vs.7.26% and no reperfusion in 7.82% vs.7.32%. Thrombolysis was given in the CCU/ICU rather than EMS or ER in 97.26% in Egypt vs.43.77% in other countries.

**Fig. 2:**
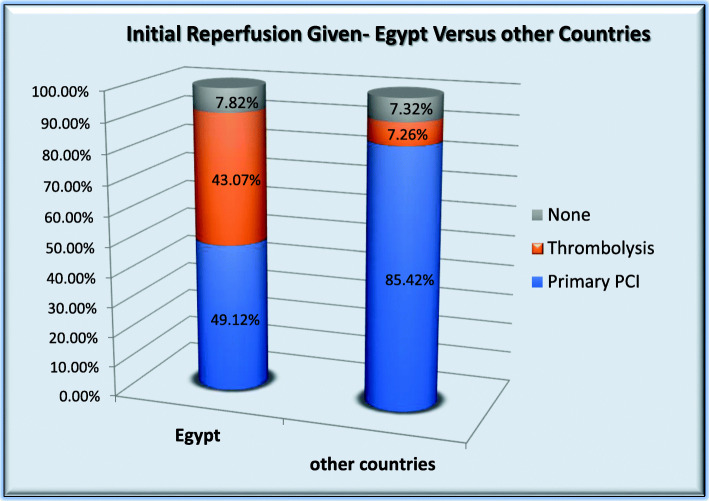
Initial reperfusion given in Egypt vs. other countries

Intended treatment for STEMI in Egypt vs. other countries was primary PCI in 50.59% vs. 85.50%, thrombolysis in 43.14% vs. 5.55%, and no reperfusion in 6.05% vs. 4.72%. Actual initial reperfusion in Egypt vs. other countries: primary PCI in 49.12% vs. 85.42%, thrombolytic therapy 43.07% vs.7.26% and no reperfusion in 7.32% vs.7.82%. Thrombolysis was given in the CCU/ICU rather than EMS or ER in 97.26% in Egypt vs. 43.77% in other countries.

### Complications during hospitalization (Table 3)

Compared to thrombolytic therapy, patients who were treated with primary PCI had less incidence of cerebrovascular accident (0.75% vs. 1.37%; *p* value < 0.001), heart failure (9.46% vs. 10.79%; *p* value < 0.001); Killip class IV (3.00% vs. 5.99%; *p* value < 0.001), atrial fibrillation (2.55% vs. 3.60%; *p* value 0.008); and no difference in mechanical complications (0.45% vs. 0.34%; *p* value 1.000). On the other hand, primary PCI had higher definite stent thrombosis (1.36% vs. 0.00%) and most serious bleeding (5.71% vs. 3.25%, *p* value 0.042).

**Table 3:** Patients course during hospitalization; Egypt vs. other countries

	Egypt (*n* = 1356)	Other countries (*n* = 7420)	*p* value
Last LV ejection fraction before discharge mean (±Std)	50.4 (± 9.6)	47.4 (± 10.4)	< 0.001 (S)
Most serious bleeding	84/1356 (6.19%)	350/7414 (4.72%)	0.001(S)
Any transfusion	6/1356 (0.44%)	115/7418 (1.55%)	0.001(S)
Cerebrovascular accident	19/1356 (1.40%)	73/7418 (0.98%)	0.166(NS)
Hemorrhagic cerebrovascular accident	4/1356 (0.29%)	16/7418 (0.22%)	0.460(NS)
Staged PCI	168/1337 (12.57%)	1481/6859 (21.59%)	< 0.001(S)
CABG	26/1355 (1.92%)	186/7418 (2.51%)	0.194(NS)
Re-infarction	11/1356 (0.81%)	101/7418 (1.36%)	0.097(NS)
Definite stent thrombosis	9/1350 (0.67%)	93/7396 (1.26%)	< 0.001(S)
Mechanical complications	5/1356 (0.37%)	65/7418 (0.88%)	0.053(NS)
Heart failure	151/1356 (11.14%)	2043/6967 (29.32%)	< 0.001(S)
Atrial fibrillation	47/1356 (3.47%)	677/7417 (9.13%)	< 0.001(S)
ECG rhythm AF at discharge	15/1293 (1.16%)	247/6700 (3.69%)	< 0.001(S)

Compared to other countries, STEMI Egyptian patients had more cerebrovascular accident (1.40% vs.0.98%; *p* value 0.166) but less re-infarction (0.81% vs. 1.36%; *p* value 0.097), stent thrombosis (0.67% vs. 1.26%; *p* value < 0.001), atrial fibrillation (3.47% vs. 9.13%; *p* value < 0.001, heart failure (11.14% vs. 29.32%; *p* value < 0.001), and higher LVEF before discharge 50.4 ± 9.6 vs. 47.4 ± 10.4; *p* value < 0.001.

### In-hospital mortality and status at discharge (Fig. 3)

In-hospital mortality was 4.65% in Egypt (vs. 3.50% in other countries; *p* value 0.040): 18.87% in no-reperfusion Egyptian patients, 2.10% in primary PCI, 4.97% in thrombolysis (*p* value < 0.001). Time between symptoms onset and death (minutes) was 2517.6 ± 3477.2 in Egypt (vs. 8558.7 ± 13826.5 in other countries; *p* value < 0.001): 1889.5 (± 2803.3) in no-reperfusion, 3701.3 ± 5403.6 in primary PCI, 2379.3 ± 2623.3 in thrombolysis; *p* = 0.532. Time between first medical contact and death (min) mean ± SD was 2301.7 ± 3461.0 in Egypt (vs. 8273.7 ± 13767.1 in other countries; *p* value < 0.001): 1652.5 ± 2867.7 in no-reperfusion, 3477.0 ± 5303.9 in primary PCI, 2182.1 ± 2622.3 in thrombolysis. Time between arrival at hospital and death or discharge (days) Mean ± SD was 3.6 ± 8.9 in Egypt (vs. 7.2 ± 7.0 in other countries, *p* value < 0.001): 2.9 ± 2.1 in no-reperfusion, 3.8 ± 12.5 in primary PCI, 3.4 ± 1.8 in thrombolysis, *p* value < 0.001. Percent of patients transferred to other hospitals if discharged alive was 47/1293 (3.63%) in Egypt vs. 1069/7026 (15.21%) in other countries *p* value < 0.001.

**Fig. 3:**
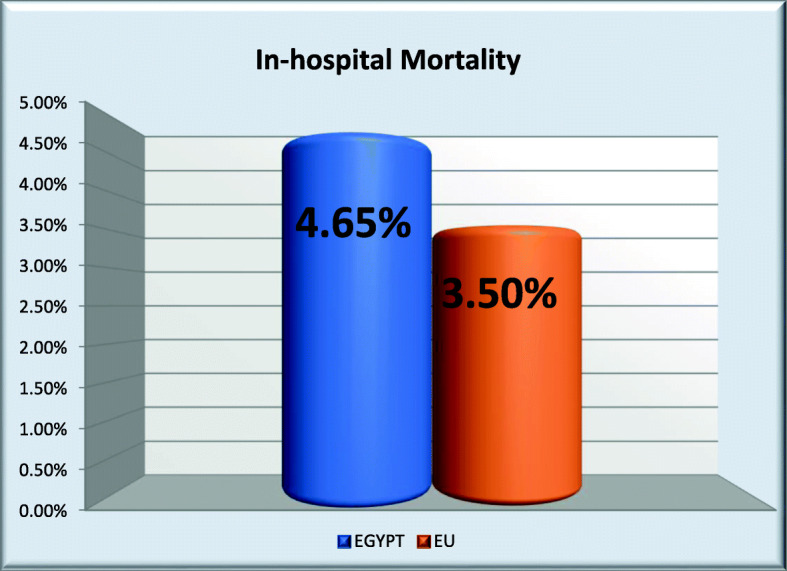
In-hospital mortality in Egypt vs. other countries

### Medications (Fig. 4)

Among Egyptian patients, the following antithrombotic agents were prescribed during first 24 h after admission: unfractionated heparin in 31.19%, low molecular weight heparin in 72.35%, and fondaparinux in 0.22%. The following medications were prescribed at discharge: aspirin in 98.61%, clopidogrel in 91.07%, ticagrelor in 7.31% and DAPT in 97.69%, aspirin and VKA and ADP receptor blockers in 26/1299 (2.00%), statins in 99.77%, beta blockers in 82.83%, ACE inhibitors in 84.76%, ARBs in 3.31%, MRAs in 10.01%, digoxin in 0.85%, ivabradine in 6.62%, ezetimibe in 1.92%, and PPIs in 48.96%.

**Fig. 4:**
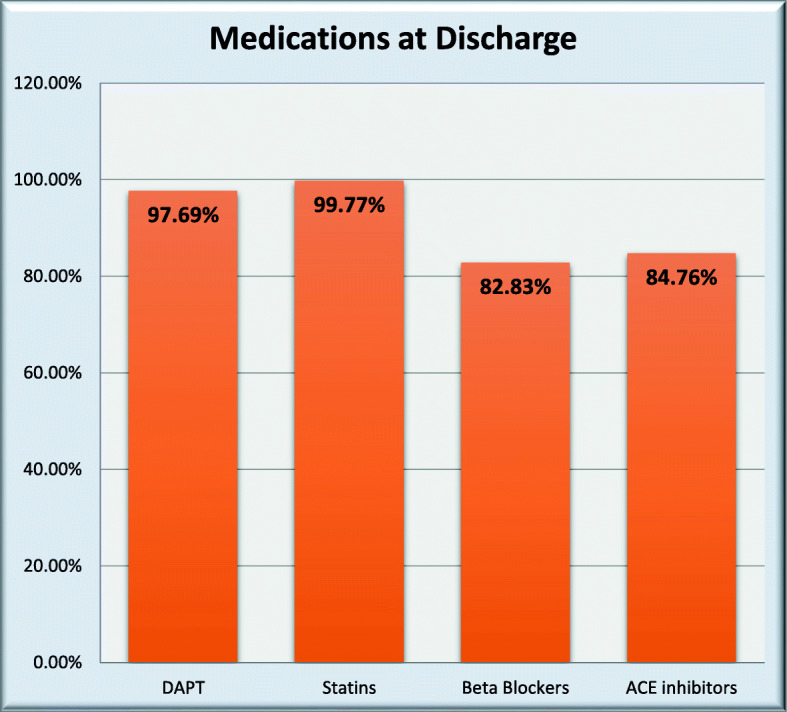
Medications at discharge among Egyptian patients

## Discussion

### Patients’ characteristics

Egyptian STEMI patients were younger than their counterparts in other countries. This observation has been reported repeatedly in many previous Egyptian studies [4]. This might be due to younger age of the whole Egyptian population, the higher prevalence and poor control of risk factors or to a more aggressive nature of atherosclerosis causing earlier coronary artery disease among Egyptians. Reports from India and other developing countries have similar observations [6, 7].

The higher prevalence of traditional risk factors among Egyptian patients highlights the importance of focusing on primary prevention which is as important as treating patients with acute cardiovascular events. Egypt needs a national primary prevention strategic plan to early detect and properly control these risk factors.

### Hospital admission process

EMS service is very much under used among Egyptian patients with STEMI. This very low rate of EMS usage among STEMI patients is similar to the lowest rate among European countries which, in one report, ranged from 18 to 85% [8].

In this registry, all indicators of out of hospital delay, like the median time between symptoms onset and call for medical help and the median time between symptoms onset and first medical contact, were significantly longer among Egyptian patients. Self-presentation rather than EMS-presentation, having hospital ER rather than the ambulance as the first medical contact, and presenting to a non-PCI capable hospital were potential causes for pre-hospital delay in Egypt [9].

In this registry, the median time in minutes between symptoms onset and call for medical help in Egypt was 85.0 vs. 74.5 in EU countries, while the median time between symptoms onset and first medical contact was 120 vs.100 min. Among patients treated with primary PCI, the median time from symptom onset to first medical contact ranged from 60 to 210 min, the time between FMC to balloon ranged from 60 to 177 min while the total ischemic time ranged from 180 to 325 min.

In an older European report, among patients treated with thrombolytic therapy, the median time from symptom onset to first medical contact ranged from 68 to 210 min, the time between FMC to thrombolytic therapy ranged from 30 to 110 min while the total ischemic time ranged from 113 to 320 min.

In an Egyptian registry, among 137 STEMI patients treated with primary PCI in a single tertiary center, the mean time of pain to FMC was 378 min while the door to balloon time was 41 min [10].

Many reports from both developed and developing countries have shown marked improvement (reduction) of total ischemic time and prehospital time delay after adopting national plans to increase patient awareness and to connect cardiac centers and EMS via regional networks [11–14].

### Patient presentation and initial assessment

The lower incidence of out of hospital cardiac arrest in Egypt might be due to either under reporting because of culture issues in Egypt to avoid post mortem investigation and the very low rate of EMS use or a true lower incidence due to younger age and lower risk profile of the Egyptian patients.

In Egypt, therapeutic hypothermia is not a well-established used technique among post-cardiac arrest patients and hypothermia when used was only external cooling pads, blankets, or wraps.

Atrial fibrillation and heart failure were lower among Egyptian patients probably due to the younger age or to under-diagnosis

### STEMI reperfusion pattern

The practice of primary PCI in Egypt has improved along the past decade despite a lot of obstacles mainly limited public medical insurance coverage and limited number of 24/7 cath labs with well trained staff especially in remote Egyptian areas. In a registry of the largest two cardiology centers in Egypt, from 2007 to 2011, only 7.2% of PCI procedures were for non-elective patients with STEMI and NSTEM [15].

In the Egyptian National heart Institute Registry, among patients with ST-elevation, 65.5% received streptokinase while only 12.4% were referred for primary PCI [16]. In a more recent registry, the rate of primary PCI among STEMI patients was 37% and thrombolysis in 54.7% [4]. The ACCESS registry, a prospective observational multinational registry, 134 sites in 19 countries in Latin America, middle east, north, and south Africa, 9732 ACS patients with 1 year follow-up, 45% STEMI and 52% NSTEMI, STEMI had fibrinolysis in 30%, and primary PCI in 26% [17].

The ACCESS registry, a prospective observational multinational registry, 134 sites in 19 countries in Latin America, middle east, north, and south Africa, 9732 ACS patients with 1 year follow-up, 45% STEMI and 52% NSTEMI, STEMI had fibrinolysis in 30% and primary PCI in 26% [17].

Among 20 European countries, there was large difference in the rate and pattern of STEMI reperfusion: primary PCI ranged from 5 to 92%, thrombolysis ranged from 1 to 55%, and no-reperfusion ranged from 7 to 52% in some countries. Egypt was not included in that report.

In a more recent report, the rate of primary PCI per 1 million inhabitants ranged from 25 to 884. The recommended figure is > 600 PPCI per 1 million inhabitants; for Egypt it was 29 per 1 million inhabitants. Egypt short-term target should be 400 PPCI per million, i.e., total of 40000 PPCI/year, i.e., 40% of the estimated total number of 100,000 STEMI/year [18, 19].

According to a recent report, Egypt has < 10 interventional cardiologist/million inhabitants compared to Germany which has ≥ 25, < 2 cath labs/million vs. ≥ 5 in Germany, and < 1 PPCI cath lab/million vs. ≥ 4 in Germany. The number of PPCI procedures was < 200 per million compared to ≥ 600 in Germany [20].

Non-reperfused patients are mainly those with late presentation beyond the window of reperfusion. Their number was probably under estimated because we included 19 very active centers that apply guidelines recommendations in the diagnosis and management of STEMI patients.

Thrombolysis is still a very common reperfusion method among STEMI patients in Egypt and is mostly given in the ICU/CCU of the hospitals and almost never in the EMS (out of hospital) nor in the ER. The thrombolytic agent given is mostly streptokinase and not TNK or rTPA due to limited resources. Routinely sending patients post thrombolysis to primary PCI within 2–24 h is generally underused strategy. Egypt should improve all aspects of this mode of reperfusion when applied.

### Complications during hospitalization

The highest incidence of complications was among the non-perfused patients, who had the highest incidence of in-hospital mortality compared to re-perfused patients.

Primary PCI patients had fewer incidences of cerebrovascular accidents heart failure and atrial fibrillation probably due to higher incidence of successful reperfusion while they had higher incidence of serious bleeding (probably related to vascular access site bleeding) and stent thrombosis obviously as stenting is their default treatment.

The higher incidence of cerebrovascular accidents among Egyptian Compared to EU patients is probably due to higher use of thrombolytic therapy among the former. The lower incidence of re-infarction, stent thrombosis, AF, and Heart failure among Egyptian patients might be underestimated or due to younger and less risk profile of these patients

### In-hospital mortality and status at discharge

The statistically significant higher in-hospital mortality among Egyptian patients despite their younger age and lower risk profile (4.65% vs. 3.50%) is probably due to the lower rate of primary PCI for reperfusion, the longer delay before first medical contact and the less application of advanced techniques like LV assisting devices and hypothermia.

In an older report, the in-hospital mortality in STEMI patients in different European countries ranged from 3 to 13%. In an American report, the in-hospital mortality for STEMI patients treated with PPCI varied between 3.1 and 6.1%. Egypt is to be compared to the lower range of these old reports, but mortality rate has already improved in these countries.

The mortality rate among Egyptian primary PCI patients reported in this registry (2.10%) indicates improvement compared to an older study (Yehia et al. 2010) in which the in-hospital mortality rate among 137 patients treated with primary PCI in one Egyptian tertiary center was 6.57%.

The highest rate of in-hospital mortality among the group of no-reperfusion (18.87%) indicates that we should encourage their earlier presentation because late presentation was the main cause of not offering them any kind of reperfusion.

### Medications

Clopidogrel is the most commonly used ADP receptor blocker even among STEMI patients (91.07%) while ticagrelor or prasugrel were only used in 7.31% of Egyptian patients. This might be due either to financial reasons where clopidogrel is available in generic forms, the higher rate of thrombolytic therapy usage where clopidogrel is the default drug, concerns about risk of bleeding or just practice inertia. More potent antiplatelets should be the default drugs used in STEMI patients according to the guidelines [21].

Statins are prescribed for most STEMI patients at discharge (99.77%), but we do not know which doses are prescribed for such high risk patients. We lack long-term follow-up studies to document the rate of patients’ compliance and adherence.

### Gaps in guideline implementation in Egypt and implications for quality improvement (Table 4)

Total ischemic time is much increased due to both patients and system delays. There is an urgent need for public awareness and patient education campaigns to instruct patients with chest pain to seek medical advice as early as possible together with improving the EMS performance in Egypt. The vast majority of patients with STEMI in Egypt have been self-presented to hospitals rather than via EMS system. We need to improve the performance of EMS in Egypt to become a dependable and efficient mode of STEMI patients transfer.

**Table 4:** Summary of possible gaps and obstacles and the suggested actions needed to improve STEMI management in Egypt

Gaps and obstacles in STEMI management in Egypt	Actions needed to optimize STEMI management in Egypt
Patients with high prevalence of risk factors	Primary prevention plan
Increased ischemic time and delayed presentation	Public awareness campaigns and patient education
Under use of EMS	Improving EMS service quantitatively and qualitatively
Under use of primary PCI	Increased health care expenditure Increase number of PPCI centers Establish STEMI networks and protocols Extend medical insurance Expand reimbursement to PPCI centers Educate and train medical personnel
Streptokinase is still widely used	Replace it with TNK Encourage out of hospital thrombolytic therapy Encourage routine PCI post thrombolysis
Clopidogrel is widely used	Encourage more potent antiplatelets
High in-hospital mortality rate	Reduce the number of non-reperfused patients Registries and quality control

The number of primary PCI/ per million populations is small due to the limited number of primary PCI centers which, when present, is not connected to a regional network of referring hospitals. There is an obvious unmet need to develop such hub and spoke system in Egypt especially in remote and rural areas. The government should extend the umbrella of medical insurance and its reimbursement to health care centers providing emergency STEMI reperfusion [22–24].

One of the main obstacles that prevent big hospitals at some times to admit patients with STEMI is the lack of empty beds in the CCU. A solution to this problem is patient repatriation after the primary PCI procedure to the local referring hospital. In our study, there was a lower incidence of patients discharged to another hospital, compared to European countries, which is due to absence of repatriation policy.

Pharmaco invasive approach might be a practical way of treating STEMI patients in remote and rural areas, in major cities with heavy traffic and in areas with inefficient EMS system. Thrombolytic agent should be the TNK and not the streptokinase and better given before reaching hospital. Routine coronary angiography with possible PCI to the infarct related artery within 2–24 h after thrombolytic therapy is a guidelines class IA recommendation but in Egypt it is often either neglected or performed after the recommended time window. Such procedure is not reimbursed by the government assuming that the patient has been already reperfused by the thrombolytic therapy [25–29].

### Limitations of this study

Despite every effort to include many centers with diverse activities and geographic locations these centers, and these patients might not represent the actual Egyptian practice especially in some remote and rural areas in Egypt.

## Conclusion

Compared to EU countries, Egyptian STEMI patients were younger, more frequently current smokers and diabetics, and had longer time between symptoms onset and first medical contact with more self-presentation rather than EMS presentation. Thrombolytic therapy is still a common reperfusion therapy in Egypt while primary PCI was offered to half of the patients. In-hospital mortality was significantly higher in Egypt and was highest among no reperfusion patients and lowest among PPCI patients. DAPT and statins at discharge were adequately following guideline recommendations.

## Data Availability

The datasets used and/or analyzed during the current registry are available from the corresponding author on reasonable request.

## Abbreviations

STEMI: ST elevation myocardial infarction
ESC: European Society of Cardiology
EMS: Emergency medical system
ECG: Electrocardiography
PCI: Percutaneous coronary intervention
EU: European Union
DAPT: Dual antiplatelet therapy
ACE: Angiotensin converting enzyme
MRAs: Mineralo receptor antagonists
PPCI: Primary percutaneous coronary intervention
CVD: Cardiovascular disease
ACCA: ACCA-EAPCI Acute Cardiovascular Care Association
EAPCI: European Association of Percutaneous Cardiovascular Interventions
EORP: EURObservational Research Programme
CCU: Coronary care unit
ICU: Intensive care unit
ER: Emergency room
LVEF: Left ventricular ejection fraction
ARBs: Angiotensin receptor blockers
FMC: First medical contact
NSTEMI: Non ST elevation myocardial infarction
ACS: Acute coronary syndrome
